# Bidirectional perspectives on internet-related family communication: Development and validation of the parent–child internet communication scale (PCICS) and associations with problematic internet use

**DOI:** 10.1016/j.abrep.2026.100734

**Published:** 2026-08-08

**Authors:** Nobuaki Morita

**Affiliations:** Institute of Medicine, University of Tsukuba

**Keywords:** Parent–child communication, Problematic internet use, Behavioral addiction, Parental mediation, Scale development, Family processes

## Abstract

This study developed and validated the Parent–Child Internet Communication Scale (PCICS), a dyadic measure assessing internet-related communication between parents and adolescents. Drawing on parental mediation theory (Livingstone & Helsper, 2008) and Schaefer's (1965) two-dimensional model of parent–child relationships (acceptance–rejection; psychological autonomy–control), items were generated to capture whether internet-related concerns can be openly shared and constructively discussed within the family. Existing measures have focused mainly on mediation behaviors or general communication quality without capturing bidirectional dyadic perceptions.

Data were collected from 824 parent–adolescent pairs via a web-based survey in Japan. Factor analyses supported a multidimensional structure: the child version (PCICS-C) yielded two factors (Parental Acceptance, Low Perceived Barriers to Consultation), and the parent version (PCICS-P) yielded three, adding Dialogic Parental Involvement. Metric invariance was confirmed across informants for the two shared subscales, and reliability was satisfactory throughout.

Concurrent validity was supported by associations with problematic internet use (PIU) and attachment security. Child-reported low perceived barriers to consultation were negatively associated with PIU, suggesting that adolescents' sense of being able to discuss internet concerns openly may lower PIU risk. Parental acceptance, however, showed weaker associations with adolescent PIU than child reports, suggesting parents' subjective sense of acceptance does not always translate into adolescent-perceived support.

These findings underscore the value of assessing internet-related family communication from both perspectives. The PCICS was designed to measure open, bidirectional communication and facilitate constructive family dialogue in intervention contexts; the observed perception gaps illustrate one clinically meaningful application of this dyadic approach.

## Highlights

• The Parent–Child Internet Communication Scale (PCICS) assesses open, bidirectional communication about internet use.
• Parallel parent and child forms enable comparison of communication perceptions within dyads.
• Child-reported low perceived barriers to consultation were linked to lower problematic internet use.
• Parental acceptance showed weaker links to adolescent PIU than child-reported measures, suggesting perception gaps.
• The PCICS offers a structured basis for facilitating constructive family dialogue in clinical settings.

## 1 Introduction

In recent years, problematic internet use among adolescents has become an increasing concern, and attention has been drawn to the importance of the home environment—particularly parent–child relationships—as factors contributing to its development and persistence. Problematic internet use has increasingly been conceptualized within a behavioral addiction framework, emphasizing its similarities to substance-related disorders in terms of compulsive use, impaired control, and functional impairment. Previous studies have highlighted the role of family dynamics in adolescent problematic internet use, with parental monitoring and communication identified as key factors influencing both risk and protective processes (Lam et al., 2009; Stattin & Kerr, 2000). A systematic review further demonstrated that positive parenting styles are negatively associated with problematic internet use, whereas negative parenting styles are positively associated (Niu et al., 2023).

Problematic internet use (PIU) during adolescence is strongly intertwined with underlying psychosocial difficulties. Because adolescents are still developing self-regulatory skills while placing high value on peer relationships, online approval and connections frequently serve as powerful psychological rewards. Consequently, the internet is often used as a coping mechanism to alleviate stress, loneliness, and social anxiety, which can escalate into uncontrolled use and subsequent symptoms such as depression, anxiety, and sleep disturbances (Caplan, 2003; Kuss & Lopez-Fernandez, 2016). In this context, parent–child communication may function as a key protective relational resource: by providing a safe space for adolescents to discuss their online experiences and concerns, open communication with parents may help buffer the transition from internet use as a coping strategy to problematic, uncontrolled use.

In understanding adolescent PIU, the family environment plays a crucial role. A meta-analysis demonstrated that parental monitoring and warmth decrease the risk of PIU, whereas conflict, rejection, and psychological control increase it (Shek et al., 2018). Furthermore, from a developmental psychopathology perspective (Bronfenbrenner, 1979), behavior problems and family dynamics form a chain of interactions. A bidirectional vicious cycle often emerges: an adolescent's excessive internet use prompts increased parental restriction, scolding, and mutual distrust, leading to a breakdown in communication, which in turn diminishes the adolescent's emotional secure base and exacerbates the excessive use. Taken together, these findings suggest that parent–child communication should be conceptualized primarily as a *protective factor*—a relational resource associated with lower PIU risk—rather than as a mediator between specific stressors and PIU outcomes.

Within this context, research on parental mediation—defined as strategies parents use to regulate and contextualize their children's media use, including active mediation (discussing content with the child), restrictive mediation (setting rules about use), and co-use (using media together; Livingstone & Helsper, 2008)—has examined how these strategies are associated with distinct developmental outcomes (Kuldas et al., 2021; Nikken & Jansz, 2014).

However, existing measures have primarily focused on behavioral aspects of parental involvement and do not adequately capture whether parents and adolescents can openly share internet-related worries and difficulties.

Several recent scales have attempted to address aspects of this limitation. For example, the Parent–Adolescent Communication about Adolescents' Social Media Use Scale (PACAS) assesses bidirectional communication processes, including disclosure and concealment (Beyens et al., 2024). However, it focuses primarily on communication structure and openness and does not sufficiently address whether problems and difficulties can be constructively shared. Similarly, the Parent–Child Communication Quality Scale (PCCQS) evaluates qualitative aspects such as empathy and acceptance (Lyu et al., 2024), but it does not directly assess problem-focused communication in the context of internet use.

In addition, prior research has often relied on reports from either parents or adolescents, limiting the ability to capture discrepancies between informants. Such discrepancies are not merely measurement error but may reflect meaningful aspects of family dynamics (Chen et al., 2023; De Los Reyes, 2013). In particular, differences between parents' perceptions of communication and adolescents' actual experiences may be important for understanding problematic internet use.

Taken together, existing measures do not fully capture whether internet-related concerns can be mutually recognized and constructively discussed within the parent–child relationship, nor do they adequately address discrepancies between parent and child perspectives. Furthermore, general parent–child communication scales are insufficient for capturing the specific dynamics of internet-related communication. Internet use involves unique contextual features—including the invisibility of adolescents' online activities to parents, the stigma associated with problematic use that may inhibit disclosure, and the rapid evolution of platform-specific behaviors—that make it a domain warranting dedicated measurement tools. A scale designed specifically for internet-related communication can more sensitively capture the barriers and facilitators of help-seeking that are particular to this context.

To address these limitations, the present study aimed to develop the Parent–Child Internet Communication Scale (PCICS) using parent–child dyadic data. The PCICS was designed to assess whether internet-related worries and problems can be shared and discussed constructively, while also capturing both shared and discrepant perceptions between parents and adolescents. In addition to its research utility, the scale was intended as a practical tool for identifying communication difficulties and facilitating dialogue in family-based prevention and intervention contexts.

To examine these communication dynamics, the present study specifically focused on high school students, corresponding approximately to late adolescence (ages 15–18; Sawyer et al., 2018). We adopt school-stage terminology rather than age-band terminology because, in the Japanese context, transitions in parental behavioral control are tied more closely to school-level transitions than to chronological age per se; for example, increased unsupervised smartphone access and reduced direct parental oversight typically coincide with the transition into high school. This developmental stage represents a critical transitional period (Sawyer et al., 2018). Compared to junior high school students, high schoolers possess greater autonomy in their internet use, predisposing them to frequent late-night usage and SNS-related interpersonal stress (Nesi et al., 2018). Conversely, unlike college students, they typically still reside at home, meaning that parental involvement remains a significant environmental factor. During this stage, parental influence shifts from direct behavioral monitoring to communication-based negotiation and rule-setting (Steinberg, 2001). Therefore, late adolescence provides an optimal developmental context for capturing the complex nuances, difficulties, and perceptual discrepancies in parent-child communication about internet use.

## 2 Method

### 2.1 Participants

Participants were recruited nationwide through a major online research agency in Japan, which maintains a pre-registered panel of households. Panel members meeting the study's eligibility criteria were identified from agency records and invited via the agency's standard notification system to participate in a dyadic parent–adolescent survey. Interested households accessed the survey through a unique link tied to their registered household account, which subsequently enabled verification of parent–child pairing (see Ethical Considerations for the consent and administration sequence). The agency was responsible for participant recruitment, screening, and incentive distribution; the research team had no direct contact with participants and received only de-identified response data. All participants were Japanese nationals residing in Japan; all survey materials were administered in Japanese. The sample consisted of 824 pairs of high school students and their biological parents. To ensure the generalizability of the findings and prevent regional bias, data were collected from all eight major geographic regions across Japan (Hokkaido to Kyushu). The inclusion criteria for the dyadic sample were as follows: (1) the parent and the high school-aged child must live together in the same household; (2) they must be biologically related; (3) both must use the internet regularly; and (4) both must agree to participate in the study. All data were collected between February 20 and March 2, 2026. Participant characteristics are summarized in Table 1, including parent and child gender, parental age, education level, employment status, and geographic distribution (e.g., Kanto 34.6%, Kinki 19.3%, Chubu 18.9%).

**Table 1:** Participant characteristics.

**Variable**	**Category**	***n***	**%**
Parent gender	Male	492	59.7
	Female	332	40.3
Parent age	30s	16	1.9
	40s	369	44.8
	50s	391	47.5
	60s and older	48	5.8
Child gender	Male	472	57.3
	Female	347	42.1
	Other (prefer not to answer)	5	0.6
Employment status	Full-time employee	554	67.2
	Stay-at-home parent	114	13.8
	Part-time, temporary, or other	150	18.2
	Unemployed	6	0.7
Education	Junior high school graduate	12	1.5
	High school graduate	185	22.5
	Junior college or vocational school graduate	203	24.6
	College or graduate school graduate	424	51.5
Region	Hokkaido	31	3.8
	Tohoku	46	5.6
	Kanto	285	34.6
	Chubu	156	18.9
	Kinki	159	19.3
	Chugoku	57	6.9
	Shikoku	26	3.2
	Kyushu	64	7.8

To conduct exploratory and confirmatory factor analyses using independent samples, participants were randomly divided into two groups (Group 0: *n* = 436; Group 1: *n* = 388) using SPSS-generated random numbers. No significant differences in demographic characteristics were found between the groups Table 2.

**Table 2:** Comparison of participant characteristics between Group 0 and Group 1.

Variable	Category	Group 0 n (%)	Group 1 n (%)	*p*-value
Parent gender	Male	261 (59.9)	231 (59.5)	ns
	Female	175 (40.1)	157 (40.5)	
Parent age	30s	6 (1.4)	10 (2.6)	ns
	40s	198 (45.4)	171 (44.1)	
	50s	214 (49.1)	177 (45.6)	
	60s+	18 (4.1)	30 (7.7)	

Note: Fisher's exact test, ns = not significant.

### 2.2 Measure development

Item generation was informed by two complementary theoretical strands, each mapped to specific factors of the scale. First, parental mediation theory (Livingstone & Helsper, 2008; Nikken & Jansz, 2014), which distinguishes active mediation, restrictive mediation, and co-use, informed items describing parents' proactive, initiative-taking engagement with their child's internet use; these items subsequently formed the Dialogic Parental Involvement factor in the parent version. Second, Schaefer's (1965) two-dimensional model of parent–child relationships—foundational to widely used instruments such as the Parental Bonding Instrument (Parker et al., 1979)—distinguishes an acceptance–rejection dimension from a psychological autonomy–control dimension. The acceptance–rejection dimension, reflecting parental warmth, understanding, and non-judgmental responsiveness, informed items that subsequently formed the Parental Acceptance factor in both versions. The autonomy–control dimension, reflecting the degree to which a parent is experienced as intrusive or controlling rather than respecting the child's autonomy, informed items assessing adolescents' willingness or hesitation to consult parents about internet-related concerns, on the rationale that a controlling or intrusive parenting style raises the perceived cost of disclosure; these items subsequently formed the Low Perceived Barriers to Consultation factor in both versions. This rationale is complemented by child disclosure theory (Stattin & Kerr, 2000), which describes the behavioral distinction between adolescents' voluntary disclosure and active concealment that these items were designed to capture. In constructing the items, we aimed to capture multiple aspects of communication, including parental involvement, adolescents' disclosure and concealment, and the quality of communication such as responsiveness and acceptance. In addition to these theoretical considerations, particular attention was paid to the potential practical usefulness of the scale in family support and intervention settings. The items were therefore designed not only to assess general communication patterns but also to capture whether internet-related anxieties, worries, and problems can be shared and discussed constructively between parents and adolescents. The intention was to develop a scale that could help identify points of difficulty in communication and serve as a starting point for facilitating dialogue about problematic internet use. Items were newly generated rather than adapted from existing measures, as no existing scale simultaneously assessed both parent and child perspectives on internet-specific communication with a help-seeking focus. Item wording was further informed by the principal investigator's clinical experience in family support settings.

We distinguish two types of constructs within the PCICS. We refer to Parental Acceptance and Low Perceived Barriers to Consultation as shared constructs: parallel items were developed for both the child and parent versions, and these constructs were subsequently confirmed to be conceptually equivalent across informants through measurement invariance testing (see Results). In contrast, we refer to Dialogic Parental Involvement as an informant-specific component: this item set was developed only for the parent version to capture parents' self-reported proactive behaviors (e.g., initiating discussions, setting rules), for which no directly parallel adolescent-report items were retained after factor analysis.

Furthermore, to capture the interactive nature of parent–child communication, parallel forms were constructed for parents and adolescents, allowing the same constructs to be assessed from both perspectives. This dyadic design makes it possible to examine both shared perceptions and discrepancies in communication experiences within the family.

The initial item pool consisted of 12 items for the child version and 20 items for the parent version, covering parental acceptance, ease of consultation regarding problems, concealment tendencies, and communication about internet use. Participants rated their responses on a 5-point Likert scale, ranging from 1 (does not apply) to 5 (applies). A multi-informant approach was adopted, and parallel items were constructed to assess the same constructs from both parent and child perspectives. Content validity was evaluated by three experts (two clinical psychologists specializing in addiction and one family therapist), who rated each item for content relevance, conceptual clarity, and practical utility using a structured rating form. Items with low expert agreement or ambiguous content were revised or eliminated prior to the factor analytic phase. No formal qualitative interview study was conducted prior to item generation; however, this is acknowledged as a limitation of the current scale development process. Items were coded such that higher scores consistently indicated higher levels of the construct. Reverse-coded items were recoded prior to analysis (see Appendix for item details).

Items were subsequently screened based on theoretical relevance and statistical criteria. Specifically, items were judged as lacking sufficient quality and removed if they met any of the following criteria: (1) low factor loadings (< 0.40) in exploratory factor analysis; (2) substantial cross-loadings (≥ 0.30 on a secondary factor); or (3) limited conceptual clarity, defined as items that were ambiguous in meaning (i.e., plausibly interpretable as measuring more than one construct) or flagged as unclear or redundant by at least two of the three expert raters. These criteria were applied iteratively, resulting in the final factor structures presented in Table 3, Table 4.

**Table 3:** Factor Loadings for the Child Version of the Parent–Child Internet Communication Scale (PCICS-C).

	Factor 1: Parental acceptance	Factor 2: Low perceived barriers to consultation
Q1	0.896	0.002
Q2	0.860	−0.003
Q3	0.808	−0.002
Q4	0.678	0.036
Q5	0.077	0.778
Q6	−0.177	0.688
Q7	0.039	0.652
Q8	0.107	0.402

Note: The number of factors was determined using principal axis factoring, based on eigenvalues ≥1 and inspection of the scree plot. Promax rotation was performed. The cumulative variance explained was 54.8%.

**Table 4:** Factor Loadings for the Parent Version of the Parent–Child Internet Communication Scale (PCICS-P).

	Factor 1: Dialogic parental involvement	Factor 2: Parental acceptance	Factor 3: Low perceived barriers to consultation
Q1	0.783	0.024	0.018
Q2	0.760	−0.013	−0.088
Q3	0.710	0.061	0.043
Q4	0.633	−0.041	−0.048
Q5	0.625	0.071	0.091
Q6	−0.046	0.878	0.037
Q7	−0.029	0.857	−0.039
Q8	0.079	0.696	−0.075
Q9	0.111	0.676	0.034
Q10	−0.008	0.079	0.840
Q11	−0.018	0.019	0.743
Q12	0.016	−0.145	0.648

Note: The number of factors was determined using principal axis factoring, based on eigenvalues ≥1 and inspection of the scree plot. Promax rotation was performed. The cumulative variance explained was 57.3%.

### 2.3 Measures for assessing concurrent validity

The Diagnostic Questionnaire (DQ; Young, 1998) is a scale designed to measure tendencies toward internet addiction. The scale consists of eight items based on the DSM-IV diagnostic criteria for pathological gambling. Respondents answer each item with “yes” or “no”; those who answer “yes” to five or more items are classified as having internet addiction, those who answer “yes” to three or four items are at risk, and those who answer “yes” to two or fewer items are classified as non-addicted. The validity of this scale for assessing adolescent PIU has been supported by subsequent psychometric studies (e.g., Beard & Wolf, 2001). The DQ was selected for concurrent validity testing because it is the most widely used PIU screening tool in Japanese adolescent research, allowing direct comparison with prior studies, and because PIU represents the primary clinical outcome of interest for the PCICS. In the present study, the standard Japanese translation of the DQ was used, which has been widely employed in Japanese PIU research (e.g., Mihara et al., 2016).

The Experiences in Close Relationships—Relationship Structures questionnaire (ECR-RS; Fraley et al., 2011) assesses attachment quality in specific interpersonal relationships (e.g., mother, father, partner, close friend). It comprises two subscales across nine items rated on a 7-point Likert scale. The Anxiety subscale assesses fear of abandonment and concerns about the availability of attachment figures (e.g., “I worry that this person won't care about me as much as I care about them”). The Avoidance subscale assesses discomfort with closeness and dependence on others (e.g., “I prefer not to share my feelings with this person”). The ECR-RS was selected because attachment security is theoretically linked to openness of communication and help-seeking behavior—core constructs of the PCICS—and prior research has demonstrated that secure attachment is associated with greater parental disclosure and consultation willingness in adolescents (Allen, 2008; Armsden & Greenberg, 1987). In the present study, attachment to both mothers and fathers was assessed using the Japanese version validated by Komura et al. (2016).

### 2.4 Statistical analysis

For exploratory factor analysis (EFA), data from Group 0 were used. Principal axis factoring was employed, and the number of factors was determined based on the criterion of eigenvalues greater than or equal to 1 and scree plots, followed by Promax rotation. Items were retained if their factor loadings were 0.40 or higher and they did not show high loadings on multiple factors; items that did not meet these criteria were removed.

Confirmatory factor analysis (CFA) was conducted using data from Group 1 to test the model. Model fit was evaluated using CFI, TLI, RMSEA, and SRMR.

Concurrent validity was examined through correlations with problematic internet use (DQ) and attachment to parents (ECR-RS).

The PCICS was designed to enable discrepancy analysis through its parallel structure. Although direct analysis of parent–child discrepancy scores is beyond the scope of the present scale development study, the parallel design allows for the computation of informant discrepancy scores (e.g., Gap = *Z*-score ∼ parent∼ − *Z*-score ∼ child∼) and mean-level composite scores (e.g., Mean = [*Z*-score ∼ parent∼ + *Z*-score ∼ child∼] / 2) following the framework proposed by De Los Reyes (2013). Such analyses represent a promising direction for future research.

### 2.5 Ethical considerations

Prior to the survey, informed consent was obtained using a rigorous two-step web-based procedure designed specifically for dyadic data collection involving minors. First, parents were provided with a comprehensive explanation of the study's purpose, the voluntary nature of participation, data security, and the guarantee of anonymity. Parents then checked a designated box on the screen to provide explicit consent for both their own participation and their high school-aged child's participation.

Following the completion of the parent's portion of the questionnaire, on-screen instructions explicitly directed the parent to hand the device to their child and to step away so they could not observe the adolescent's responses, thereby ensuring the child's privacy. The adolescent was then presented with an age-appropriate explanation of the study and indicated their assent by proceeding to answer their portion of the survey. Parent-child pairing was verified through the use of a single authenticated household account registered with the survey agency. The study was conducted with the approval of the first author's institutional Ethics Committee (Approval No. 2236).

All scale items are presented in Appendix A (child version) and Appendix B (parent version).

## 3 Results

(1) Factor Structure

Exploratory factor analysis (EFA) was conducted separately for the child and parent versions of the PCICS.

For the child version (PCICS-C), a two-factor structure was identified (Table 3). The first factor, labeled *parental acceptance*, consisted of items reflecting parents' understanding and non-judgmental listening toward children's experiences related to internet use. The second factor, labeled *low perceived barriers to consultation*, included items representing children's hesitation or difficulty in discussing internet-related issues with their parents.

For the parent version (PCICS-P), a three-factor structure was identified (Table 4). The first factor, *dialogic parental involvement*, comprised items reflecting proactive engagement in discussions about internet use, including addressing potential problems and sharing perspectives. The second factor, *parental acceptance*, represented parents' attitudes of understanding and accepting their children's thoughts and feelings. The third factor, *low perceived barriers to consultation*, reflected parents' perceptions of their children's ease in seeking help and discussing concerns.

**Fig. 1:**
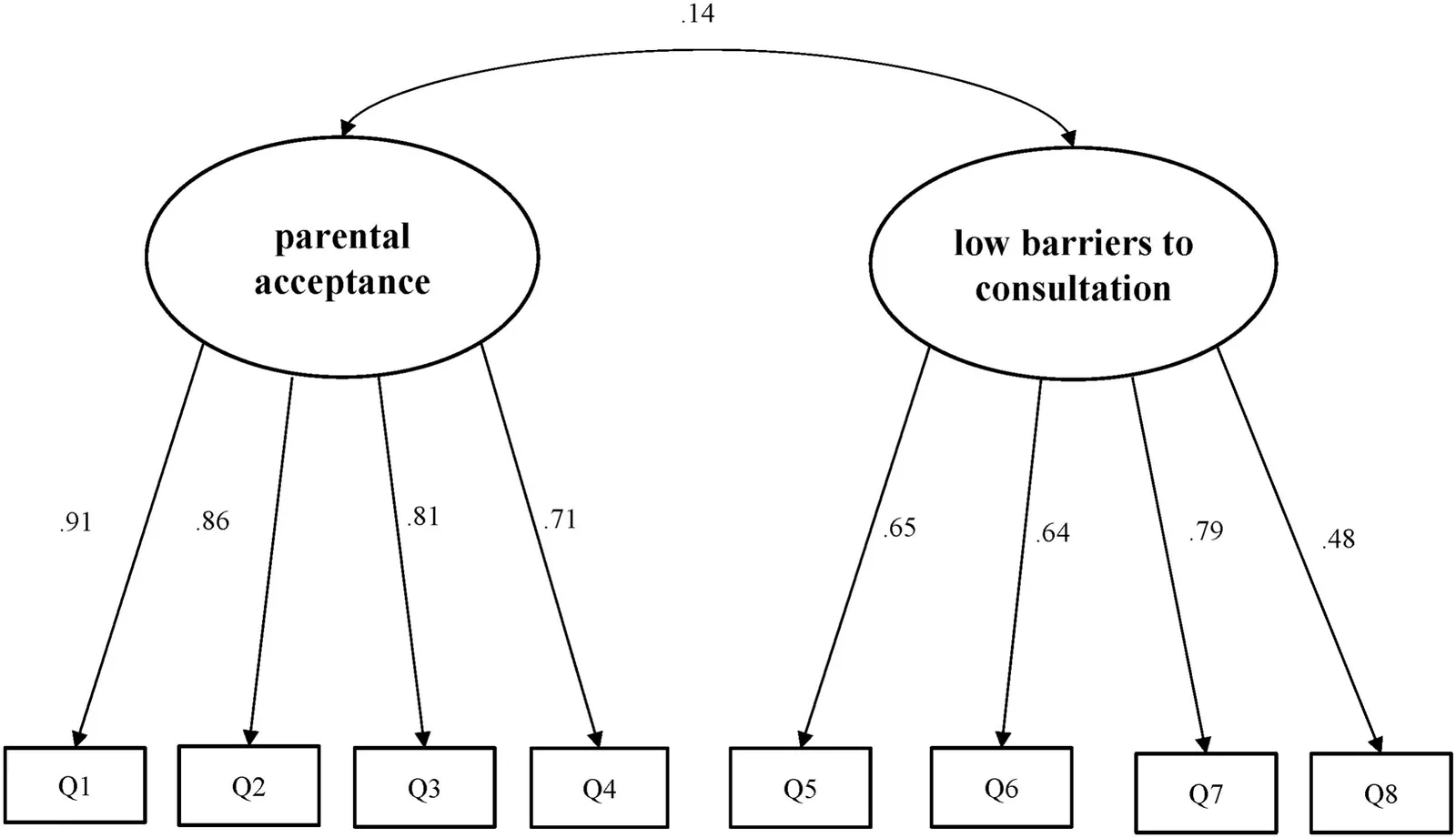
Confirmatory factor analysis model for the child version of the Parent–Child Internet Communication Scale (PCICS-C). Standardized estimates are shown. Model fit indices were as follows: CFI = 0.965, TLI = 0.949, RMSEA = 0.079, and SRMR = 0.072.

**Fig. 2:**
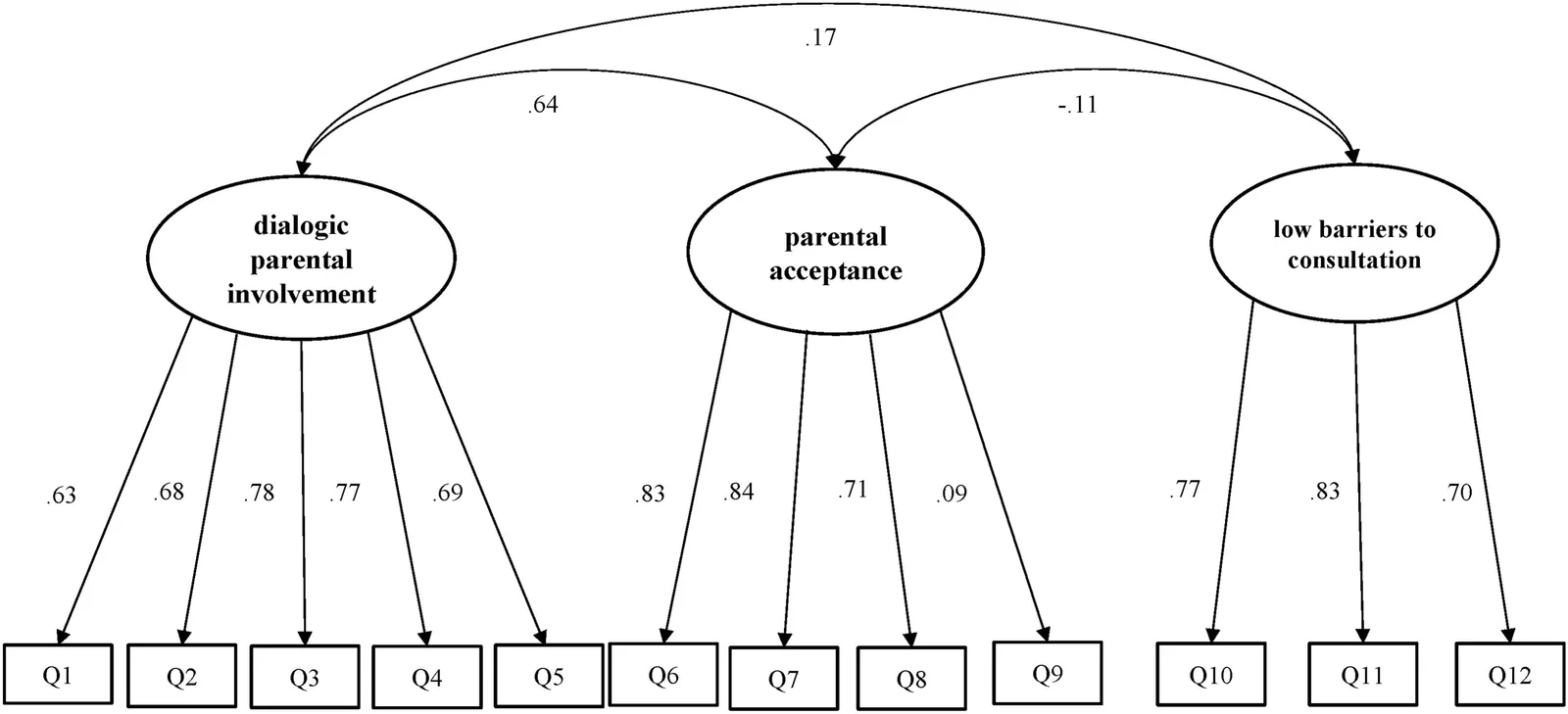
Confirmatory factor analysis model for the parent version of the Parent–Child Internet Communication Scale (PCICS-P). Standardized estimates are shown. Model fit indices were as follows: CFI = 0.969, TLI = 0.960, RMSEA = 0.052, and SRMR = 0.049.

Confirmatory factor analysis (CFA) supported these structures. The PCICS-C showed acceptable model fit (CFI = 0.965, TLI = 0.949, RMSEA = 0.079, SRMR = 0.072; Fig. 1), and the PCICS-P demonstrated good model fit (CFI = 0.969, TLI = 0.960, RMSEA = 0.052, SRMR = 0.049; Fig. 2).

To ensure that the scale scores were comparable across informants, we tested measurement invariance across parents and children using multi-group confirmatory factor analysis (MGCFA). For this analysis, we extracted a core parallel subset of six items (three for Parental Acceptance and three for Low Perceived Barriers to Consultation) that were conceptually identical across both versions. As shown in Table 5, the configural model demonstrated adequate fit, establishing that the fundamental factor structure is equivalent across informants. Subsequently, the metric invariance model was tested by constraining factor loadings to be equal across groups. The changes in fit indices (ΔCFI = −0.002, ΔRMSEA = −0.006) were well within the recommended thresholds (ΔCFI ≥ −0.010, ΔRMSEA ≤0.015; Chen, 2007), providing strong support for metric invariance. Although exact scalar invariance was not supported, achieving metric invariance confirms that the constructs have the same meaning for both parents and adolescents, justifying the comparison of their associations with other variables.

(2) Reliability

**Table 5:** Measurement Invariance of the Core Parallel Items Across Parents and Adolescents.

Model	χ^2^	df	RMSEA	SRMR	CFI	TLI	ΔCFI	ΔRMSEA
Configural	102.46***	16	0.081	0.049	0.978	0.958	–	–
Metric	113.28***	20	0.075	0.050	0.976	0.964	−0.002	−0.006
Scalar	177.83***	24	0.088	0.057	0.960	0.951	−0.016	0.013

*Note*. *N* = 1648 (824 dyads). RMSEA = root mean square error of approximation; SRMR = standardized root mean square residual; CFI = comparative fit index; TLI = Tucker-Lewis index. ΔCFI and ΔRMSEA represent the change in fit compared to the preceding, less constrained model. ***p < .001.

The Cronbach's alpha coefficients for the PCICS-C were 0.89 for Factor 1, 0.73 for Factor 2, and 0.73 for the total score. For the PCICS-P, the alpha coefficients were 0.84 for Factor 1, 0.87 for Factor 2, and 0.78 for Factor 3, with a total score of 0.84. These results indicate that both versions of the scale demonstrated adequate internal consistency.

(3) Concurrent validity

To examine the concurrent validity of the PCICS-C, correlation analyses were conducted with scores from existing measures (Table 6). The results showed that most subscales of the PCICS were negatively correlated with problematic internet use and attachment-related variables. However, several associations were weak or nonsignificant, and some were in the unexpected direction. The pattern of associations varied across subscales and informants, suggesting that each factor may reflect distinct aspects of parent–child communication. These findings are discussed in relation to the multidimensional structure of the scale in the Discussion section.

**Table 6:** Correlations of PCICS Scores With DQ and Attachment Variables.

Scale	Subscale	Parent DQ Score	Child DQ Score	Avoidance (mother)	Anxiety (mother)	Avoidance (father)	Anxiety (father)
PCICS-C	Parental acceptance	−0.139***	−0.140***	−0.492***	−0.308***	−0.410***	−0.302***
	Low perceived barriers to consultation	−0.258***	−0.346***	0.119***	−0.386***	0.144***	−0.372***
	Total score	−0.256***	−0.310***	−0.293***	−0.458***	−0.217***	−0.446***
PCICS-P	Dialogic parental involvement	−0.120***	−0.105**	−0.327***	−0.273***	−0.275***	−0.276***
	Parental acceptance	−0.058	0.042	−0.316***	−0.253***	−0.247***	−0.252***
	Low perceived barriers to consultation	−0.253***	−0.169***	0.042	−0.325***	0.074*	−0.288***
	Total score	−0.176***	−0.164***	−0.325***	−0.381***	−0.252***	−0.370***

Note. Pearson correlations. * *p* < .05, ** *p* < .01, *** p < .001. Avoidance and anxiety refer to attachment dimensions measured by the ECR-RS.

## 4 Discussion

The present study developed and validated the Parent–Child Internet Communication Scale (PCICS) using parent–child dyadic data. The findings support a multidimensional structure of communication regarding internet use and highlight both shared and informant-specific components across parent and child perspectives.

This study extends existing parental mediation and family communication frameworks by introducing a dyadic measure with two primary aims: to assess open, bidirectional communication about internet-related concerns, and to provide a practical tool for facilitating constructive family dialogue. Specifically, whereas the PACAS (Beyens et al., 2024) captures the structural openness of communication—that is, whether disclosure or concealment occurs—without directly assessing perceived acceptance, and the PCCQS (Lyu et al., 2024) evaluates general relational quality across communication domains, the PCICS was designed to assess a narrower, help-seeking-oriented construct specific to internet use: whether concerns can be recognized and constructively discussed. This distinction is reflected empirically in the present findings, where low perceived barriers to consultation—rather than general communication warmth—showed the strongest associations with adolescent PIU. Perception gaps between informants represent one clinically meaningful application of this broader approach. It should be emphasized that the present study focused on the development and validation of the PCICS itself; empirical analyses of parent–child discrepancy scores were not conducted here and are proposed as a discrete direction for subsequent research using this instrument.

Specifically, parental acceptance and low perceived barriers to consultation emerged as common dimensions across both versions of the scale, suggesting that these factors represent core elements of supportive communication. Parental acceptance reflects an empathic and non-judgmental stance, which has been consistently associated with adolescents' openness and psychological adjustment. Low perceived barriers to consultation indicate the extent to which adolescents can seek support without hesitation, which may facilitate help-seeking behaviors in the context of problematic internet use.

In contrast, dialogic parental involvement emerged only in the parent version. This finding is theoretically interpretable: parents may represent their own communication behavior in terms of active, initiative-taking practices—such as initiating discussions, setting rules, and addressing problems—whereas adolescents evaluate the same interactions primarily in terms of relational outcomes, namely whether they feel accepted and whether they can seek help without hesitation. This pattern is consistent with research on parent–child perception gaps demonstrating that parents and adolescents tend to weight different aspects of the same interactions (Smetana, 2010), and reflects a broader difference in how communicative roles are cognitively represented across generations. Importantly, the two constructs shared across both versions—parental acceptance and low perceived barriers to consultation—provide a conceptually meaningful basis for cross-informant comparison.

Specifically, the lack of a significant correlation between parental reports of acceptance and adolescents' problematic internet use (DQ) warrants clinical attention. This discrepancy suggests that while parents may perceive themselves as being accepting and non-judgmental, such internal attitudes might not be effectively communicated to the child or may not be sufficient to buffer the onset of problematic internet use. In clinical settings, this highlights the importance of moving beyond simply encouraging an ‘accepting attitude’ in parents; instead, interventions should focus on the behavioral manifestation of that acceptance—how it is actually perceived and experienced by the adolescent.

This informant discrepancy is consistent with prior research documenting differences between parent and child perceptions of family processes, and highlights the importance of assessing communication from both perspectives. From a clinical perspective, such perception gaps may represent key targets for intervention, as reducing these discrepancies could improve communication effectiveness and reduce risk behaviors.

The patterns of concurrent validity were generally consistent with theoretical expectations, although several associations were weak, non-significant, or in the opposite direction. Rather than undermining the validity of the scale, these findings may reflect the complexity of parent–child communication. In particular, some communication behaviors may operate as reactive processes that emerge in response to existing difficulties. For example, increased parental involvement or reduced perceived barriers to consultation may occur when adolescents are already experiencing problematic internet use, thereby attenuating or reversing simple linear associations.

In addition, discrepancies between parent and child reports may contribute to these findings, as each version of the PCICS captures subjective experiences from different perspectives. Such informant discrepancies have been widely documented and are increasingly recognized as meaningful indicators of relational dynamics rather than mere measurement error. The present findings underscore the importance of conceptualizing parent–child communication as an interactive and clinically relevant process in the context of behavioral addiction.

Discrepancy scores derived from the PCICS may have particular clinical relevance. Prior research has consistently demonstrated that informant discrepancies in family communication are associated with adolescent behavioral and emotional problems (Chen et al., 2023; De Los Reyes, 2013). Systematic examination of how such discrepancies relate to PIU and other outcomes represents an important direction for future research using the PCICS.

Beyond its research utility, the PCICS may have potential practical applications. By assessing communication from both parent and adolescent perspectives in parallel, the scale enables the identification of discrepancies in how communication is experienced within the family. It should be noted, however, that normative data and clinical cutoff scores have yet to be established, and the scale should therefore be regarded as a research instrument with clinical potential rather than a standalone clinical tool. One concrete application may involve sharing parallel PCICS results within a family consultation session to help identify and discuss specific communication gaps, without implying that the scale alone is sufficient for clinical decision-making.

Several limitations should be noted. First, although RMSEA for the child version slightly exceeded conventional thresholds, other fit indices indicated acceptable model fit. Given that RMSEA is sensitive to model complexity and degrees of freedom, the overall fit of the model was considered acceptable. Second, the cross-sectional design limits causal interpretation, and longitudinal research is needed to clarify the directionality of the observed associations. Third, the sample was restricted to Japanese high school students and their parents, limiting generalizability to other developmental stages, cultural contexts, and family structures. Fourth, the study relied exclusively on self-report data, which is susceptible to social desirability bias and recall bias; observational or longitudinal designs would provide richer contextual information. Fifth, no formal qualitative methods (e.g., cognitive interviews or focus groups) were employed prior to item generation, which may limit the content validity of the scale; this is acknowledged as a limitation of the current development process. Sixth, normative data and clinical cutoff scores have not yet been established, which limits the scale's immediate applicability as a standalone clinical assessment tool. Seventh, several items in the parent version require parents to infer their child's internal states (e.g., whether the child would consult them or feels hesitant to talk), which may introduce systematic bias and complicate the interpretation of parent–child discrepancies. Finally, data were collected via an online survey platform, which may undersample families with limited digital access and thus introduce selection bias.

Despite these limitations, the present study contributes to the literature by providing a theoretically grounded and practically applicable measure of parent–child communication regarding internet use. By capturing both shared and discrepant perceptions, the PCICS offers a useful framework for understanding relational processes underlying adolescent problematic internet use.

In conclusion, the PCICS represents a novel tool for assessing parent–child communication in the context of internet use. Its dual-perspective design allows for a more comprehensive understanding of family communication and provides a basis for both research and intervention aimed at promoting healthier internet use among adolescents.

## 5 Conclusion

The present study developed and validated the Parent–Child Internet Communication Scale (PCICS), a reliable and valid measure for assessing internet-related communication between parents and adolescents. By capturing both shared and distinct dimensions across parent and child perspectives, the scale provides a useful tool for examining relational processes underlying adolescent problematic internet use.

Notably, the findings are consistent with the existence of a perception gap in parent–child interactions; while parents may perceive themselves as being accepting, such subjective attitudes were not necessarily associated with a reduction in adolescents' problematic internet use. This pattern suggests that internal parental attitudes may not always be experienced as supportive by the adolescent, though longitudinal research is needed to clarify the directionality of this relationship.

Beyond its research utility, the PCICS may also have potential applications in family-based prevention and intervention contexts. By providing parallel assessments from both parents and adolescents, the scale offers a structured basis for identifying perceptual discrepancies and facilitating communication-focused dialogue. Further research is needed to evaluate its practical utility in applied settings.

Funding and author contribution details have been removed from this version for blind review and are provided on the Title Page.

## CRediT authorship contribution statement

**Nobuaki Morita:** Writing – review & editing, Writing – original draft, Validation, Resources, Project administration, Methodology, Investigation, Funding acquisition, Formal analysis, Data curation, Conceptualization.

## Funding

This work was supported by the 10.13039/501100003478Ministry of Health, Labour and Welfare, Japan (FY2025 Research Project on Addiction; Grant Number: 202605).

The author declares that there are no known competing financial interests or personal relationships that could have appeared to influence the work reported in this paper.

## Declaration of competing interest

The author declares no competing interests.

## Data Availability

Data will be made available on request.

## Appendix A Items of the Parent–Child Internet Communication Scale (Child Version; PCICS-C)

Q1. My parents understand how I feel about the internet right now (both the fun and the worries).

Q2. My parents listen carefully to what I have to say about the internet without dismissing it, right until the end.

Q3. I think I would consult my parents without hesitation if I ran into trouble online.

Q4. When setting rules for internet use or giving me advice, my parents respect my point of view and my thoughts.

Q5. (reverse-scored) I hesitate to discuss how I use the internet with my parents.

Q6. (reverse-scored) I sometimes find it hard to voice my hopes and requests regarding the internet to my parents.

Q7. (reverse-scored) I have internet-related worries (such as interpersonal relationships) that I find difficult to discuss with my parents.

Q8. (reverse-scored) When my parents are busy with work or other things, they turn a blind eye to my prolonged internet use.

## Appendix B Items of the Parent–Child Internet Communication Scale (Parent Version; PCICS-P)

Q1. When I sense a problem with my child's internet use, I do not ignore it; instead, I have a serious discussion with them until we both reach a mutual understanding.

Q2. I regularly discuss the positive and negative aspects of the internet, as well as points to be cautious about, with my child.

Q3. I have told my child that if they encounter any problems online, they should talk to me right away.

Q4. My child and I review specific online etiquette together, such as how to handle personal information and exchange messages.

Q5. When setting rules for internet use or offering guidance, I respect my child's perspective and opinions.

Q6. I listen carefully to my child's stories about their online activities without dismissing them.

Q7. I understand my child's current feelings (both enjoyment and concerns) regarding their online activities.

Q8. My child openly shares with me what happens online and how they feel about it.

Q9. If my child encounters any problems online, they will consult me without hesitation.

Q10. (reverse-scored) My child seems hesitant to discuss their internet usage with me.

Q11. (reverse-scored) My child seems to be struggling with internet-related concerns (such as interpersonal relationships) that they find difficult to discuss with me.

Q12. (reverse-scored) My child sometimes seems reluctant to share their hopes or requests regarding their online activities with me.

## Appendix A Supplementary data

Supplementary Table S1. Original item pool and elimination rationale for the Parent–Child Internet Communication Scale (PCICS). This table lists all initially generated items for both the child and parent versions of the PCICS, indicating which items were retained in the final scale (with their assigned factor) and the reasons for elimination of excluded items.
