# Supplementary material for: Bidirectional perspectives on internet-related family communication: Development and validation of the parent–child internet communication scale (PCICS) and associations with problematic internet use
Source: Addict Behav Rep. 2026 Aug 8;24:100734. doi: 10.1016/j.abrep.2026.100734 (PMC13495092; doi:10.1016/j.abrep.2026.100734)
Supplement: Supplementary file 1 [file mmc1.docx]

**Supplementary Table S1**

**Original Item Pool and Elimination Rationale for the Parent–Child Internet Communication Scale (PCICS)**

| **Child Version (PCICS-C): Initial Item Pool (12 items) → Retained Items (8 items)** | | | |
| --- | --- | --- | --- |
| **Item No.** | **Item Text** | **Retained Factor** | **Reason for Elimination** |
| 1 | I talk openly with my parents about what happens online and how I feel. | — | Substantial cross-loading on multiple factors |
| 2 | My parents listen carefully to what I have to say about the internet without dismissing it, right until the end. | *Parental Acceptance* | — |
| 3 | My parents understand how I feel about the internet right now (both the fun and the worries). | *Parental Acceptance* | — |
| 4 | I think I would consult my parents without hesitation if I ran into trouble online. | *Low Perceived Barriers to Consultation* | — |
| 5* | I sometimes find it hard to voice my hopes and requests regarding the internet to my parents. | *Low Perceived Barriers to Consultation* | — |
| 6* | I have internet-related worries (such as interpersonal relationships) that I find difficult to discuss with my parents. | *Low Perceived Barriers to Consultation* | — |
| 7* | I hesitate to discuss how I use the internet with my parents. | *Low Perceived Barriers to Consultation* | — |
| 8* | When my parents are busy with work or other things, they turn a blind eye to my prolonged internet use. | — | Low factor loading (< .40); limited conceptual clarity (reflects parental monitoring behavior rather than communication quality) |
| 9 | My parents regularly talk to me about the pros and cons of the internet, as well as what I need to be careful about. | — | Substantial cross-loading on multiple factors |
| 10 | When setting rules for internet use or giving me advice, my parents respect my point of view and my thoughts. | *Parental Acceptance* | — |
| 11 | When my parents think there's a problem with how I use the internet, they don't just ignore it—they try to have a serious talk with me. | — | Low factor loading (< .40) |
| 12 | My parents go over online etiquette with me, such as how to share personal information and how to communicate through messages. | — | Low factor loading (< .40) |
| **Parent Version (PCICS-P): Initial Item Pool (20 items) → Retained Items (12 items)** | | | |
| **Item No.** | **Item Text** | **Retained Factor** | **Reason for Elimination** |
| 1 | My child openly shares with me what happens online and how they feel about it. | *Parental Acceptance* | — |
| 2 | I listen carefully to my child's stories about their online activities without dismissing them. | *Parental Acceptance* | — |
| 3 | I understand my child's current feelings (both enjoyment and concerns) regarding their online activities. | *Parental Acceptance* | — |
| 4 | If my child encounters any problems online, they will consult me without hesitation. | *Low Perceived Barriers to Consultation* | — |
| 5* | My child sometimes seems reluctant to share their hopes or requests regarding their online activities with me. | *Low Perceived Barriers to Consultation* | — |
| 6* | My child seems to be struggling with internet-related concerns (such as interpersonal relationships) that they find difficult to discuss with me. | *Low Perceived Barriers to Consultation* | — |
| 7* | My child seems hesitant to discuss their internet usage with me. | *Low Perceived Barriers to Consultation* | — |
| 8 | For children, the internet is an essential space for expanding and maintaining friendships. | — | Low factor loading (< .40); items appeared to reflect adolescents' stress-related internet use motivations and parental beliefs about internet use rather than communication processes per se |
| 9 | For children, the internet has become an important way to relieve daily stress and anxiety. | — | Low factor loading (< .40); reflected adolescents' stress-related motivations and parental beliefs rather than communication processes |
| 10 | I believe children today face intense stress and anxiety that they have no choice but to relieve online. | — | Low factor loading (< .40); reflected parental beliefs about adolescent stress rather than communication quality |
| 11 | For children, the internet is an indispensable tool for obtaining information and getting things done. | — | Low factor loading (< .40); reflected beliefs about internet utility rather than communication processes |
| 12 | Enjoying games and videos online has become a source of daily vitality (the will to live) for children. | — | Low factor loading (< .40); reflected parental beliefs about internet use rather than communication processes |
| 13 | I regularly discuss the positive and negative aspects of the internet, as well as points to be cautious about, with my child. | *Dialogic Parental Involvement* | — |
| 14 | When setting rules for internet use or offering guidance, I respect my child's perspective and opinions. | *Dialogic Parental Involvement* | — |
| 15 | When I sense a problem with my child's internet use, I do not ignore it; instead, I have a serious discussion with them until we both reach a mutual understanding. | *Dialogic Parental Involvement* | — |
| 16 | I have told my child that if they encounter any problems online, they should talk to me right away. | *Dialogic Parental Involvement* | — |
| 17* | If I scold my child for using their phone or playing video games, they get upset, so even if I think it's dangerous, I end up letting them do it anyway. | — | Substantial cross-loading; limited conceptual clarity (reflects conflict avoidance rather than communication quality) |
| 18* | When problems arise over phone or video game use, I end up taking my frustration out on my child. | — | Substantial cross-loading; limited conceptual clarity (reflects parental emotional dysregulation rather than communication) |
| 19* | When I'm busy with work or other things, I turn a blind eye to my child spending long hours online. | — | Substantial cross-loading; limited conceptual clarity (reflects parental monitoring rather than communication) |
| 20 | My child and I review specific online etiquette together, such as how to handle personal information and exchange messages. | *Dialogic Parental Involvement* | — |

*Note. Items marked with an asterisk (*) are reverse-scored. EFA = Exploratory Factor Analysis. Items were eliminated if they met one or more of the following criteria: (1) low factor loading (< .40) in EFA; (2) substantial cross-loading (≥ .30 on a secondary factor); (3) limited conceptual clarity, defined as items flagged as ambiguous or redundant by at least two of three expert raters. Retained items are listed as they appear in the final scale (Appendix A and B of the main manuscript).*
